# Validity of Self-Reported Hypertension: Findings from the Thai Cohort Study Compared to Physician Telephone Interview

**DOI:** 10.5539/gjhs.v6n2p1

**Published:** 2013-11-10

**Authors:** Prasutr Thawornchaisit, Ferdinandus de Looze, Christopher M Reid, Sam-ang Seubsman, Adrian Sleigh, The Thai Cohort Study Team

**Affiliations:** 1 School of Medicine, University of Queensland, Australia; 2 School of Public Health and Preventive Medicine, Monash University, Australia; 3 School of Human Ecology, Sukhothai Thammathirat Open University, Thailand; 4 National Centre for Epidemiology and Population Health, ANU College of Medicine, Biology and Environment, The Australian National University, Australia

**Keywords:** self-reported hypertension, physician telephone interview, agreement, validity, population prevalence, Thailand

## Abstract

**Background:**

Surveys for chronic diseases, and large epidemiological studies of their determinants, often acquire data through self-report since it is feasible and efficient. We examined validity and associations of self-reported hypertension, as verified by physician telephone interview among participants in a large ongoing Thai Cohort Study (TCS).

**Methods:**

The TCS investigates the health-risk transition among distance learning Open University students living all over Thailand. It began in 2005 and at 4-year follow-up, 60 569 self-reported having or not having doctor diagnosed hypertension. Two hundred and forty participants were randomly selected from each of the “hypertension” and “normotension” self-report groups. A Thai physician conducted a structured telephone interview with the sampled participants and classified them as having hypertension or normotension. The sensitivity, specificity, positive and negative predictive value (PPV and NPV) and overall accuracy of self-report were calculated.

**Results:**

The sensitivity of self-reported hypertension was 82.4% and the specificity was 70.7%. As true prevalence was simulated to vary from 1% to 50% the overall accuracy of self-report varied little from 71% to 75%. High sensitivity and negative predictive value related to female gender, younger age (≤40 years), higher education attainment and not visiting a physician in the last 12 months. High specificity and positive predictive value related to female gender, older age, higher education attainment and visiting a doctor in the previous year.

**Conclusion:**

Self-report of hypertension had high sensitivity and good overall accuracy. This is acceptable for use in large studies of hypertension, and for estimating its population prevalence to help formulate health policy in Thailand.

## 1. Introduction

Hypertension is a medical and public heath challenge around the globe (Kearney et al., 2005). Elevated blood pressure damages many organs and body systems but is most important as the key risk factor for cardiovascular disease and stroke (Chobanian et al., 2003). Ischaemic heart disease and cerebrovascular disease are ranked first and second respectively in the ten leading causes of global mortality (Lopez, Mathers, Ezzati, Jamison, & Murray, 2006) and are the top two out of three major causes of death in Thailand (Porapakkham et al., 2010). Accordingly, hypertension has been analysed in the Thai Cohort Study (TCS), a nationwide investigation developed in 2005 to analyse the transition of health risk factors and the associated emergence of chronic disease in Thailand. There were 87 134 baseline participants and the original aims and design are reported elsewhere (Sleigh, Seubsman, & Bain, 2008). A 4-year follow up study was conducted in 2009 and 60,569 responded (Seubsman, Yiengprugsawan, Sleigh, & Thai Cohort Study, 2012; Seubsman et al., 2011). An 8-year follow up is underway in 2013. Here we report our assessment of hypertension measurement in the Thai Cohort Study.

Assessment of health status, including hypertension, in large cohort studies such as TCS is made on the basis of responses to mailed, self-report questionnaires. This method is convenient and relatively inexpensive for obtaining population data and widely adopted in cohort studies across the globe. However, the accuracy of such data is a concern as reliable self-reported information is essential for research on determinants and for planning and implementation of public health policy aimed at prevention. The impact of the ongoing health risk transition driving the rapid emergence of chronic conditions in developing countries, particularly hypertension and cardiovascular disease, has major implications for a nation’s health. Self-reported data must reflect the occurrence of hypertension if they are to be a valuable tool for monitoring overall trends and for study of determinants. However, database searches confirm there are limited data on the accuracy of self-report of hypertension in Asian populations in general and Thailand in particular (Taylor et al., 2010; Wu, Li, & Ke, 2000).

Assessing the accuracy of population health information when validating epidemiological studies has mainly relied on comparing self-reported data with a “gold standard” such as medical record information or measurements from physical examinations. These methods are costly and labour intensive. Additionally, a further limitation is that most validation studies have been conducted in Western populations based on small, unrepresentative samples. This has limited their ability to identify characteristics of respondents that are associated with inaccurate reporting (Goldman, Lin, Weinstein, & Lin, 2003). Our study addresses some of these issues for hypertension, an important chronic disease now emerging in many transitional settings, including Thailand.

Published research has shown that the validity of self-report of hypertension is affected by many factors including age, gender, education attainment, income and having a recent health check-up (Goldman et al., 2003; Tormo, Navarro, Chirlaque, & Barber, 2000; Vargas, Burt, Gillum, & Pamuk, 1997). Additionally, self report may be influenced by the wording or sequence of the questionnaire as well as cognitive or situational factors (Fadnes, Taube, & Tylleskär, 2009). Recall bias may be an issue and is significantly influenced by the time between an event and its assessment (Hassan, 2006). A recent systematic review and meta-analysis of the accuracy of self-reported hypertension compared with blood pressure measurement showed that the prevalence of hypertension is under-estimated and that awareness is low and highly variable (Gorber, Tremblay, Campbell, & Hardt, 2008). This review also reported that many validation studies did not follow standard guidelines for blood pressure measurement.

We aimed to investigate the validity of self-reported hypertension in Thailand. The participants were members of the Thai Cohort Study (TCS) and they lived all over Thailand. Validation of self-reported hypertension by comparing with participants’ medical records or measuring their blood pressure was infeasible both in terms of cost and time. Furthermore, measurement of blood pressure would be uninformative among those already treated because their elevated blood pressure would usually have been normalized. Accordingly, for the validation we used a telephone based structured interview by a Thai physician as the “gold standard” for diagnosis of hypertension among the cohort, and compared the physician telephone results to self-report.

## 2. Methods

The validation study reported here was designed to provide a “gold standard” of hypertension diagnosis against which the accuracy, sensitivity, specificity and predictive values of self-report could be measured. The gold standard used physician telephone interviews and a sampling system as described below. First, the telephone interview questions and an interpretative algorithm (Addendums 1 and 2) were developed through an iterative process involving hypertension experts, an epidemiologist and a member of the TCS team. Then, over a four week period from March to April 2011, a random sample of cohort members who had responded to the TCS survey in 2009 were interviewed by telephone by a Thai physician (PT). Telephone interviewees were classified as hypertensive or normotensive in 2009 according to the algorithm and this “gold standard” result was compared to self-report.

**Addendum 1:** Physician telephone interview validation questions (2011)

1	Did you have hypertension / high blood pressure in 2009? Yes / No
	If Yes – ask questions 2 and 3 and by whom / where diagnosed
	If No – ask question 4
2	How long have you had hypertension / high blood pressure? (___ years)
3	At what age were you diagnosed with hypertension / high blood pressure?
4	Have you ever been diagnosed with hypertension / high blood pressure?
4.1	Diagnosed by physician…………. diagnosed by a nurse or others…………․
4.2	Name of hospital or clinic where diagnosed with hypertension․․․․․․․․․․․․․․․․․․․․․․․
5	Do you know your blood pressure level / measurement? Yes / No
6	What is / was your most recent blood pressure level / measurement?
	SBP………………mmHg DBP………………mmHg
7	Do you take any drug / medication for hypertension? (Yes / No)
8	Please give me the name of any / all drugs / medications you are taking.
	a……………………………………….b………………………………………….
	c……………………………………….d………………………………………….
	(Any anti-hypertensive drug - Yes / No)
9	Have you visited a physician in the last 12 months? Yes / No

**Addendum 2:** The criteria used for diagnosis of Hypertension or Normotension by physician telephone interview, numbered to correspond with interview questions

Hypertension (HT)	Normotension (NT)
1 Yes = HT	1 No = no self reported HT
	(If no self reported HT, questions 2 and 3 will be excluded)
2 known duration of HT	2 not applicable
3 age/year participant diagnosed with HT	3 not applicable
4.1 Diagnosed by physician or nurse	4.1 never diagnosed with HT
4.2 Can give name of hospital or clinic where participant was diagnosed HT	4.2 not applicable
6.1 Systolic blood pressure >140	6.1 Systolic blood pressure <140
6.2 Diastolic blood pressure>90	6.2 Diastolic blood pressure <90
7 Takes anti-hypertensive drug(s)	7 No anti-hypertensive drug(s)
9 Names any anti-hypertensive drug	8 No anti-hypertensive drug named
*Participant will be identified as having HT if he/she has at least one criterion above (other than 1).*	*Participant will be identified as having NT if giving the defined response to questions 1, 4.1, 6.1, 6.2, 7, 8.*

### 2.1 Sample Size Determination

Sample size was calculated using STATCALC software (Lee, McInerney, & Mullins, 1984). With 3,676 participants self reporting hypertension in 2009, the calculation of sample size needed for investigating this group was based on a source population of 4000. False self-report was predicted at 20% with a detected level of 15% considered to be acceptable at the 5% significance level. STATCALC estimated that 232 completed interviews would be required to attain such precision of validation among the self-reported hypertension group. A similar sample size of 232 would also be suitable for investigating the self-reported normotension group.

We expected that at least one-third of listed cohort members who we attempted to contact would be reached by telephone and would agree to the interview. So each self-report group (hypertension and normotension) would require about 696 individuals on its initial list for attempted contact. Accordingly, for each group, we randomly listed 720 TCS participants (using the random number function in SPSS (Pallant, 2011)). Contact success was as expected and we were able to complete 240 physician interviews for each self-report group (hypertension and normotension).

### 2.2 Selection of Study Samples and Interview Participants

Figure 1 shows how the 240 participants in the hypertension group were selected, balancing for age, sex, educational attainment and income. We expect that this balance allows the intrinsic properties of the self-report diagnosis to be measured without confounding. Consequently, 24 sub-groups were identified and from each of these 10 randomly selected participants were interviewed as described below until 240 completed interviews were obtained. The same selection process was used for the normotension group. Thus a total of 480 were interviewed, 240 for each of the hypertension and normotension groups.

**Figure 1:**
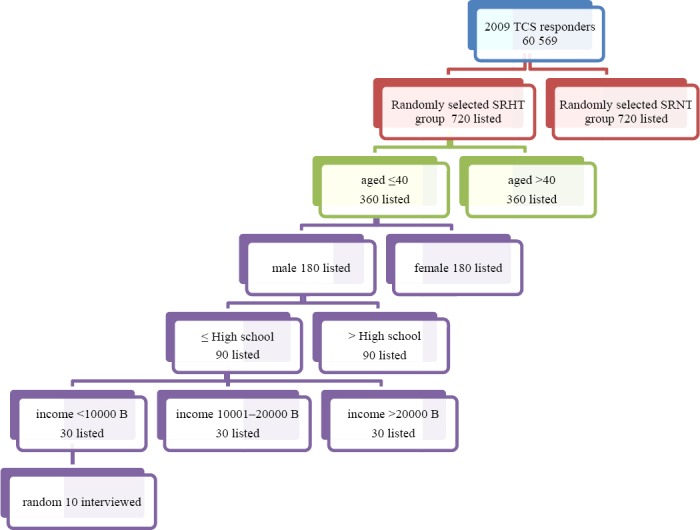
Schema for validation study subject selection (self reported hypertension group and self reported normotension group) matching for age, sex, education and income

Note: after stratifying the 60 569 cohort members by self-reported hypertension (hypertensive or normotensive), age (≤40, >40), sex, education (≤ high school, >high school) and income (three categories) the study population was divided into 24 sub-groups. Within each of these 24 sub-groups, a random list of 30 individuals was prepared and at random 10 were interviewed. Therefore, the two analysis groups (hypertension and normotension) each had 240 members selected at random and matched for age, sex, education and income.

Most listed cohort members had a mobile, home or office phone numbers. Calls were made initially to mobile phones then to home and office phones, respectively. Each number was dialed three times and if there was no answer the next number was used. If a selected participant could not be contacted within three calls, he/she was bypassed. This was continued in order of the name on the random sampling list of each sub-group until 10 participants were interviewed (Figure 1). In most of the 24 sub-groups only 15-20 participants were called to obtain the 10 interviews but in two groups all 30 were needed to successfully contact 10. Overall, less than 1 in 10 of those contacted refused to participate.

### 2.3 Statistical Analysis

SPSS was used to find Cohen’s Kappa value of agreement and its 95% confidence interval between physician interview classification and self-report of hypertension or normotension among all participants (Norusis, 2012). A p-value of 5% or less was used as the criterion for statistical significance.

The result of the physician telephone interview was used as the gold standard for calculating sensitivity, specificity, positive and negative predictive value (PPV and NPV) of self-reported hypertension. These same test parameters were also calculated after stratifying (separately) by sex, age (two groups), education (two groups) and income (three groups). These variables had previously reported as influential in self-reporting hypertension. Finally, the overall accuracy of self-reported hypertension was determined by calculating the total proportion correctly classified (ie proportion of true positives plus proportion of true negatives).

As well the performance of self-report as a function of the underlying population prevalence was simulated using the calculated sensitivity and specificity and varying the prevalence from one to fifty percent.

## 3. Results

The selection process used to obtain the validation study samples from the TCS population yielded participants with an equal distribution of the four influential variables of interest (Figure 1). At the time of interview in 2011, more than three quarters of the sample had visited a doctor within the last 12 months indicating that a similarly large proportion would be expected to have done so in 2009.

Among those who self-reported hypertension in 2009 nearly 60% confirmed this historical status on telephone interview in 2011 (Table 1). Nearly fifty percent recalled that they had been diagnosed as having hypertension by a doctor and eight percent by a nurse. Just under half (48%) were on anti-hypertensive medication, while over half (56%) knew their latest blood pressure level. Three-quarters had seen a physician in the last 12 months. Assessment of physician telephone interview responses concluded that 64% of the self-reported hypertension group did have hypertension in 2009 according to our criteria.

**Table 1:** Responses to physician telephone interview (PTI) questions by hypertension self-report (SR) group

Hypertension assessment telephone questions (PTI)*	Frequency of positive answer for PTI by SR group*			
	Hypertension group (N=240)		Normotension group (N=240)	
	n	%	n	%
Q1. Did you have HT in 2009?	141	(58.8)	7	(2.9)
Q4.1. HT Diagnosed by doctor?	118	(49.2)	Not applicable	Not applicable
Q4.2. Diagnosed by nurse?	20	(8.3)	Not applicable	Not applicable
Q5. Do you know your BP?	134	(55.8)	124	(51.7)
Q7. Do you take anti-HT drugs?	116	(48.3)	3	(1.3)
Q9. Have you visited a Physician in last 12 months	179	(74.6)	191	(79.6)
HT by validation criteria (Q1-Q7) – see Addendum 2	154	(64.2)	33	(13.8)

* SR = self-report by 2009 TCS questionnaire; PTI = physician telephone interview in 2011

In parallel, 97% of those reporting normotension in the 2009 TCS confirmed this historical status on telephone interview in 2011 (Table 1). Anti-hypertensive medication was being taken by 1.3% of this group. Half of the normotension group knew their blood pressure level and four in five had visited a physician at least once in the last 12 months. Assessment of physician telephone interview responses concluded that 86% of the self-reported normotension group were indeed normal in 2009 according to our criteria.

In our whole validation study sample (n=240 + 240) the overall prevalence of hypertension by self-report is set by design at 50%. When each individual was reassessed by physician telephone interview the overall prevalence of hypertension among the 480 sampled persons fell to 39%. Accordingly, self-report led to over-reporting of the actual prevalence by 11% (Table 2). Evidently, agreement between self-report and physician telephone interview was moderate. Indeed, Cohen’s Kappa value was 0.5 (95%CI = 0.43-0.58).

**Table 2:** Comparison of self-report to physician telephone interview for detection of hypertension in the Thai Cohort Study

Influential variables	Self-report *versus* physician telephone interview		
	Prevalence difference* (fall below 50%)	Test of Agreement	
		Kappa value (95% CI)	p-value** (Kappa)
Sex			
Male	11.6	0.4 (0.3-0.6)	<0.0001
Female	10.5	0.6 (0.5-0.7)	<0.0001
Age			
≤40	24.3	0.5 (0.4-0.6)	<0.0001
>40	-2.1	0.5 (0.4-0.7)	<0.0001
Education			
≤ High school	11.5	0.4 (0.3-0.5)	<0.0001
> High school	10.6	0.6 (0.5-0.7)	<0.0001
Personal income			
≤10000	13.6	0.5 (0.3-0.6)	<0.0001
10001–20000	12.6	0.6 (0.5-0.7)	<0.0001
>20000	6.9	0.5 (0.3-0.6)	<0.0001
Visit doctor in last 12 months			
Yes	6.2	0.6 (0.5-0.7)	<0.0001
No	27.3	0.3 (0.2-0.5)	<0.0001
Overall	11.0%	0.5 (0.4-0.6)	<0.0001

* Hypertension and normotension groups combined (n=240+240). Accordingly, self-reported hypertension is set at 50% by design. In this column, physician telephone interview prevalence is subtracted from the self-report prevalence.

** p-value for difference within the group tested by Kappa

Variable categories substantially adversely affecting validity of self-report included age less than 40 years and not visiting a doctor in the last 12 months (Table 2). Other variables tested (sex, education and income) showed little difference across categories for moderate agreement regarding hypertension status. A notable exception to this categorical indifference was high income which, unlike low and middle income, was associated with a very small difference in prevalence.

The sensitivity, specificity, PPV and NPV of self-reported hypertension are as shown in Table 3 (82.4%, 70.7%, 64.2% and 86.3%, respectively). Self-report permits 82% of “true” hypertension cases and 70% of “true” normotension cases to be identified. The overall accuracy of self report was about 75% under the conditions of this study.

**Table 3:** Sensitivity, specificity and positive or negative predictive value (PPV, NPV) of hypertension self-report as assessed by physician telephone interview

Personal attributes	Sensitivity (%)	Specificity (%)	PPV (%)	NPV (%)
Sex				
Male	78.5	67.8	60.3	83.5
Female	86.2	73.6	68.1	89.1
Age				
≤40	95.2	65.5	49.2	97.5
>40	76.0	78.5	79.2	75.2
Education				
≤ High school	78.7	67.8	60.7	83.5
> High school	86.0	73.6	67.8	89.1
Personal income				
≤10000	82.0	67.3	60.2	86.1
10001–20000	88.1	73.0	65.8	91.3
>20000	77.6	71.7	66.7	81.5
Visit doctor in last 12 months				
Yes	82.1	76.2	71.5	85.3
No	83.9	55.7	42.6	89.8
Overall	82.4	70.7	64.2	86.3

Sensitivity and NPV were noticeably higher in females, the younger age group (≤40 years), and those with higher education (Table 3). Specificity and PPV tended to increase with older age, female gender, higher educational attainment and visiting a physician at least once in the last 12 months. Consequently, female gender and having a higher educational attainment were associated with the accuracy of self-reported hypertension.

The simulated performance of self-report for diagnosis of hypertension and normotension shows that the overall accuracy (71% to 76%) was relatively stable as true prevalence varied from one percent to fifty percent (Table 4). PPV rose from 23 percent to 74 percent as NPV fell from 100% to 80% as the true prevalence increased from one percent to fifty percent. Evidently PPV was more variable than NPV and NPV was more variable than overall accuracy.

**Table 4:** Simulated positive and negative predictive values and overall accuracy for self-reported hypertension varying the true prevalence from one percent to fifty percent

True prevalence (%)	PPV (%)*	NPV (%)*	Overall accuracy (%)*
1	23	100	71
10	24	98	71
20	41	95	73
30	54	90	74
40	65	86	75
50	74	80	76

* PPV = positive predictive value; NPV= negative predictive value; overall accuracy= true positive plus true negative as a proportion of the total

## 4. Discussion

To our knowledge, this is the first report of an attempt to validate self reported hypertension through a structured questionnaire administered by a physician conducting a telephone interview (PTI) with respondents. Self-reported hypertension was moderately more frequent when compared with PTI assessment. But the concordance was acceptable and relatively stable at 71% to 76%, increasing gradually as the true prevalence was simulated to rise from one percent to fifty percent.

Over-report of hypertension was higher in those of younger age, with lower personal income and who had not visited a doctor in the last 12 months. This may be a result of white coat hypertension (Pierdomenico et al., 1995) or an error of diagnosis where it is not based on the standard criteria for hypertension (average of two blood pressure readings taken under specified conditions in each visit at two visits (Chobanian et al., 2003). Diagnosis error could be due to measurement error, a failure of doctor-patient communication or a 24-hour fluctuation of blood pressure (Taylor et al., 2010).

In our study of hypertension in a nationwide cohort of Thai adults, agreement of self-report and PTI was moderate and not affected by age, sex, educational level and personal income. By comparison, agreement was higher in a US study (Leikauf & Federman, 2009) but lower in an Australian study (Taylor et al., 2010) for younger participants. The moderate agreement seen in males and females was similar to that in a study in Taiwan (Goldman et al., 2003). Similarly, agreement was not associated with participants’ educational attainment and household income in Canadian (Muhajarine, Mustard, Roos, Young, & Gelskey, 1997) and American populations (Leikauf & Federman, 2009). However, agreement in participants who had visited a doctor at least once in the last 12 months was higher than for those who had not. This result was consistent with previous validation studies in Spanish and Taiwanese populations (Goldman et al., 2003; Vargas et al., 1997).

Overall sensitivity was high in this study and relatively stable across categories for age, sex, education and income. Sensitivity did decrease with age. This was the opposite of the findings in previous studies (Ford, Harel, Heath, Cooper, & Caspersen, 1990; Molenaar, Van Ameijden, Grobbee, & Numans, 2007; Taylor et al., 2010; Tormo et al., 2000). It was likely that young Thais who reported hypertension were diagnosed by a physician and younger people tend to have only a few health problems while older people may have many health problems. In a Dutch study, visiting health services was the most important predictor of sensitivity of self-reported hypertension (Molenaar et al., 2007).

In Thailand, people can have their blood pressure measured at local health services by health personnel at no cost. All Thais enjoy the benefits of lifespan universal health coverage (Tangcharoensathien et al., 2011; Yiengprugsawan, Seubsman, Lim, Sleigh, & Thai Cohort Study, 2009) which allows equal access to public health services free of charge. The results indicate that Thai STOU students are concerned about their health status regardless of age, sex, educational level and income. In this population, visiting a doctor was an important factor for the accuracy of self-reported hypertension.

Sensitivity of self-reported hypertension was higher in women and those with higher education. This gender difference was noted in previous studies (Ford et al., 1990; Molenaar et al., 2007; Tormo et al., 2000; Vargas et al., 1997) but some of these and other studies (Molenaar et al., 2007; Okura, Urban, Mahoney, Jacobsen, & Rodeheffer, 2004; Vargas et al., 1997) reported no sensitivity differences for participants’ education. In our study, the majority of highly educated participants were female so the higher sensitivity in better educated participants was confounded by gender. One US study in a Hispanic population found that sensitivity tended to have an inverse association with personal income (Ford et al., 1990). But in our study there was no consistent relationship between sensitivity and income.

Specificity was acceptable in this study and two thirds of those with “true” normotension could be identified by self-report. Specificity was found to be a little higher for the older group (over 40) and females and this was in contrast to findings in Spanish and US studies (Ford et al., 1990; Tormo et al., 2000). Both groups are generally more concerned with their health status and therefore more likely to visit a doctor more regularly (Taylor et al., 2010).

Determining the validity of self report in this study is dependent on the extent to which the instrument used is itself a valid measurement of the diagnosis of hypertension. How well does this instrument meet requirements for construct validity? The structured questionnaire as administered by a physician has strong face validity. The development of the questionnaire by an expert panel and the iterative process involved ensures that all relevant content domains are represented, thus conferring a high degree of content validity. Similarly, agreement by the expert panel on the pre-specified criteria required to classify an individual as hypertensive (having hypertension) or being normotensive (not having hypertension) also ensures a degree of convergent validity. On the basis of the above we make the case that administration of this structured questionnaire by PTI is a valid tool in classifying survey respondents and determining the degree of accuracy of self reported hypertension.

Recall bias is a potential limitation of the findings of the present study as two years had elapsed between the completion of the TCS follow-up in 2009 and the physician telephone interview in 2011. Recalling when a diagnosis was made is often difficult, particularly for older subjects. Also communication of a clinician’s diagnosis and the understanding of the participant as to its meaning may be a limitation. More extensive research to validate physician telephone interview assessment of doctor diagnosed hypertension against medical records would be desirable but is infeasible on any large scale in Thailand. We consider it self evident that a careful medical history by a trained physician, as used here, is going to be close to the most accurate method that can be devised to detect hypertension in a well-educated population that regularly receive medical care.

We conclude that this validation study has provided evidence that self-reported hypertension in a very large cohort yields epidemiologically useful information about that condition. Furthermore, in developing and transitioning countries where information and resources are scarce, this self-report method is suitable for the study of hypertension and population health.
